# Methyl 2,4-dihy­droxy-5-(2-methyl­propanamido)­benzoate

**DOI:** 10.1107/S1600536813000457

**Published:** 2013-01-12

**Authors:** Syeda Sohaila Naz, Nazar Ul Islam, M. Nawaz Tahir, Muhammad Raza Shah

**Affiliations:** a University of Peshawar, Institute of Chemical Sciences, Peshawar, Pakistan; b University of Sargodha, Department of Physics, Sargodha, Pakistan; c H.E.J. Research Institute of Chemistry, International Center for Chemical and Biological Sciences, University of Karachi, Karachi 75270, Pakistan

## Abstract

In the title compound, C_12_H_15_NO_5_, the dihedral angle between the benzene ring and the C atoms of the terminal isopropyl group is 83.48 (16)°. Intra­molecular N—H⋯O and O—H⋯O hydrogen bonds generate *S*(5) and *S*(6) rings, respectively. In the crystal, mol­ecules are linked by O—H⋯O hydrogen bonds, generating *C*(7) chains propagating in [001]. Weak aromatic π–π stacking [centroid–centroid separation = 3.604 (3) Å] is also observed.

## Related literature

For related structures, see: Chen *et al.* (2011 ▶); Naz *et al.* (2013 ▶).
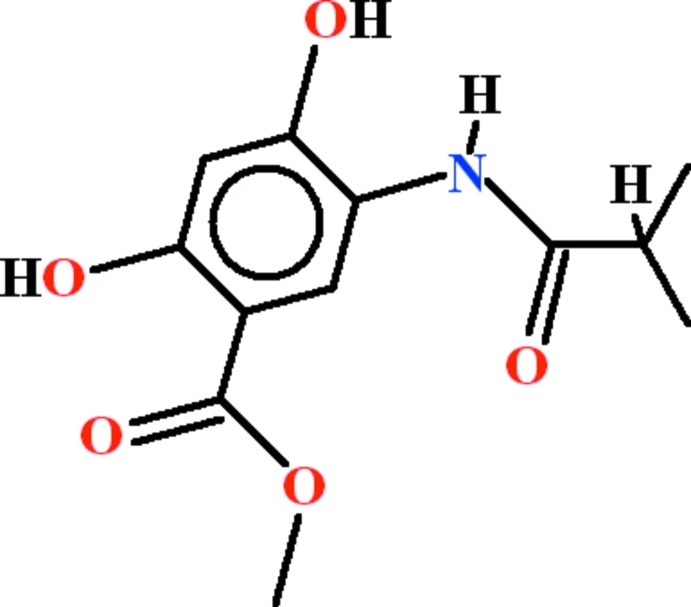


## Experimental

### 

#### Crystal data

C_12_H_15_NO_5_
*M* *_r_* = 253.25
Monoclinic, 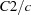

*a* = 22.732 (4) Å
*b* = 8.2338 (16) Å
*c* = 14.743 (3) Å
β = 113.506 (9)°
*V* = 2530.4 (9) Å^3^
*Z* = 8
Mo *K*α radiation
μ = 0.10 mm^−1^
*T* = 296 K
0.26 × 0.16 × 0.14 mm

#### Data collection

Bruker Kappa APEXII CCD diffractometer
Absorption correction: multi-scan (*SADABS*; Bruker, 2009 ▶) *T*_min_ = 0.981, *T*_max_ = 0.985
8480 measured reflections
2218 independent reflections
950 reflections with *I* > 2σ(*I*)
*R*_int_ = 0.090

#### Refinement

*R*[*F* ^2^ > 2σ(*F* ^2^)] = 0.065
*wR*(*F* ^2^) = 0.168
*S* = 0.96
2218 reflections
169 parameters
H-atom parameters constrained
Δρ_max_ = 0.25 e Å^−3^
Δρ_min_ = −0.19 e Å^−3^

### 

Data collection: *APEX2* (Bruker, 2009 ▶); cell refinement: *SAINT* (Bruker, 2009 ▶); data reduction: *SAINT*; program(s) used to solve structure: *SHELXS97* (Sheldrick, 2008 ▶); program(s) used to refine structure: *SHELXL97* (Sheldrick, 2008 ▶); molecular graphics: *ORTEP-3* (Farrugia, 2012 ▶) and *PLATON* (Spek, 2009 ▶); software used to prepare material for publication: *WinGX* (Farrugia, 2012 ▶) and *PLATON*.

## Supplementary Material

Crystal structure: contains datablock(s) global, I. DOI: 10.1107/S1600536813000457/hb7024sup1.cif

Structure factors: contains datablock(s) I. DOI: 10.1107/S1600536813000457/hb7024Isup2.hkl

Supplementary material file. DOI: 10.1107/S1600536813000457/hb7024Isup3.cml

Additional supplementary materials:  crystallographic information; 3D view; checkCIF report

## Figures and Tables

**Table 1:** Hydrogen-bond geometry (Å, °)

*D*—H⋯*A*	*D*—H	H⋯*A*	*D*⋯*A*	*D*—H⋯*A*
N1—H1⋯O4	0.86	2.19	2.604 (4)	109
O3—H3⋯O2	0.82	1.87	2.595 (4)	146
O4—H4⋯O5^i^	0.82	1.820	2.633 (4)	174

Symmetry code: (i) 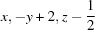.

**Fig. 1.**
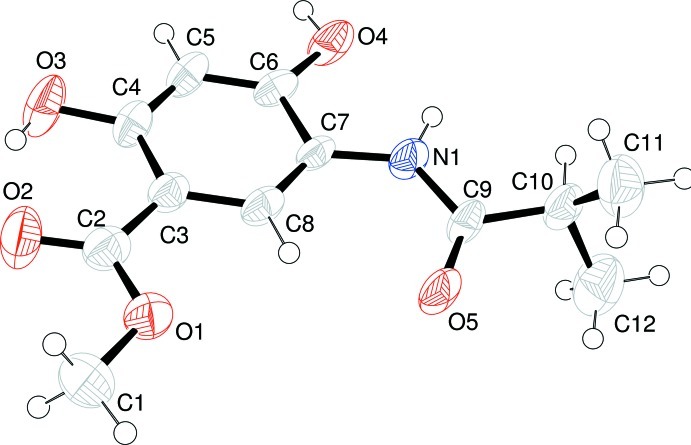
View of the title compound with displacement ellipsoids drawn at the 50% probability level.

**Fig. 2.**
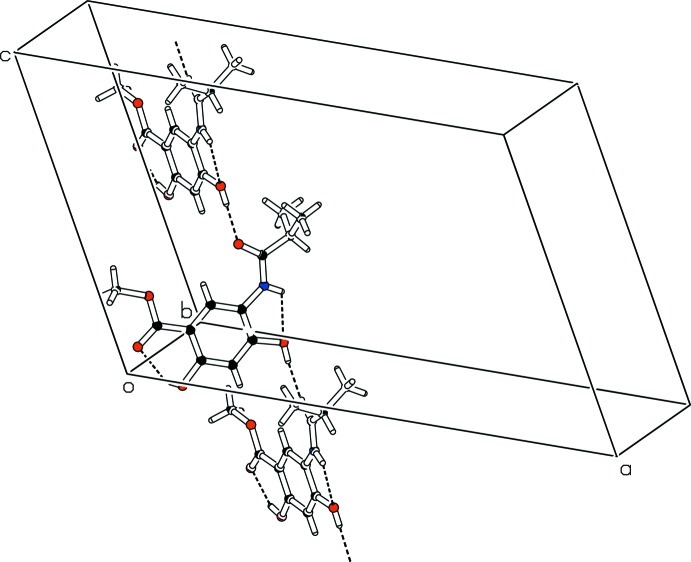
The partial packing of (I), which shows that molecules form S(5) & S(6) loops and one dimensional polymeric chains are formed due to O—H···O H-bonds along the [001] direction.

## supplementary crystallographic information

### Comment

The title compound (I, Fig. 1) has been prepared for derivatization and for the biological studies in continuation to form different derivatives of methyl 5-amino-2,4-dihydroxybenzoate (Naz *et al.*, 2013). The crystal structure of 3-hydroxy-2-(isobutyrylamino)benzamide (Chen *et al.*, 2011) has been published which is related to the title compound.

In (I), the groups A (C1—C8/O1—O4/N1) of methyl 5-amino-2,4-dihydroxybenzoate is almost planar with r. m. s. deviation of 0.0190 Å. The C9 and O5 atoms are at a distance of -0.1205 (50) and -0.3867 (44) Å from the mean square plane of the group A. The isopropyl group B (C10—C12) is of course planar. The dihedral angle between A/B is 83.24 (15)°. There exist strong intramolecular H-bondings of N—H···O and O—H···O types (Table 1, Fig. 2) completing S(5) and S(6) ring motifs. There also exist strong intermolecular H-bondings of O—H···O type due to which *C*(7) chains are formed (Table 1, Fig. 2) resulting in the formation of one dimensional polymeric network along the *c*-axis. There also exist π–π interactions between the centroids of benzene rings at a distance of 3.604 (3) Å.

### Experimental

Equivalent amounts of methyl 5-amino-2,4-dihydroxybenzoate (0.2 g, 1.1 mmol) and Isobutyric anhydride (0.2 ml, 1.1 mmol) were heated at 333 K for 3 h in dimethylformamide (DMF). The reaction mixture was kept for 48 h to afford brown needles of the title compound.

### Refinement

The H-atoms were positioned geometrically (C–H = 0.93–0.98, N—H = 0.86 and O—H = 0.82 Å) and refined as riding with *U*_iso_(H) = x*U*_eq_ (C, N, O), where x = 1.5 for hydroxy & methyl groups and x = 1.2 for all other H-atoms.

### Crystal data

**Table:**

C_12_H_15_NO_5_	*F*(000) = 1072
*M**_r_* = 253.25	*D*_x_ = 1.330 Mg m^−^^3^
Monoclinic, *C*2/*c*	Mo *K*α radiation, λ = 0.71073 Å
Hall symbol: -C 2yc	Cell parameters from 950 reflections
*a* = 22.732 (4) Å	θ = 2.0–25.0°
*b* = 8.2338 (16) Å	µ = 0.10 mm^−^^1^
*c* = 14.743 (3) Å	*T* = 296 K
β = 113.506 (9)°	Needle, brown
*V* = 2530.4 (9) Å^3^	0.26 × 0.16 × 0.14 mm
*Z* = 8	

### Data collection

**Table:**

Bruker Kappa APEXII CCD diffractometer	2218 independent reflections
Radiation source: fine-focus sealed tube	950 reflections with *I* > 2σ(*I*)
Graphite monochromator	*R*_int_ = 0.090
Detector resolution: 8.10 pixels mm^-1^	θ_max_ = 25.0°, θ_min_ = 2.0°
ω scans	*h* = −26→26
Absorption correction: multi-scan (*SADABS*; Bruker, 2009)	*k* = −9→9
*T*_min_ = 0.981, *T*_max_ = 0.985	*l* = −12→17
8480 measured reflections	

### Refinement

**Table:**

Refinement on *F*^2^	Secondary atom site location: difference Fourier map
Least-squares matrix: full	Hydrogen site location: inferred from neighbouring sites
*R*[*F*^2^ > 2σ(*F*^2^)] = 0.065	H-atom parameters constrained
*wR*(*F*^2^) = 0.168	*w* = 1/[σ^2^(*F*_o_^2^) + (0.067*P*)^2^] where *P* = (*F*_o_^2^ + 2*F*_c_^2^)/3
*S* = 0.96	(Δ/σ)_max_ < 0.001
2218 reflections	Δρ_max_ = 0.25 e Å^−^^3^
169 parameters	Δρ_min_ = −0.19 e Å^−^^3^
0 restraints	Extinction correction: *SHELXL97* (Sheldrick, 2008), Fc^*^=kFc[1+0.001xFc^2^λ^3^/sin(2θ)]^-1/4^
Primary atom site location: structure-invariant direct methods	Extinction coefficient: 0.0032 (7)

### Special details

**Table:**

Geometry. Bond distances, angles *etc*. have been calculated using the rounded fractional coordinates. All su's are estimated from the variances of the (full) variance-covariance matrix. The cell e.s.d.'s are taken into account in the estimation of distances, angles and torsion angles
Refinement. Refinement of *F*^2^ against ALL reflections. The weighted *R*-factor *wR* and goodness of fit *S* are based on *F*^2^, conventional *R*-factors *R* are based on *F*, with *F* set to zero for negative *F*^2^. The threshold expression of *F*^2^ > σ(*F*^2^) is used only for calculating *R*-factors(gt) *etc*. and is not relevant to the choice of reflections for refinement. *R*-factors based on *F*^2^ are statistically about twice as large as those based on *F*, and *R*- factors based on ALL data will be even larger.

### Fractional atomic coordinates and isotropic or equivalent isotropic displacement parameters (Å^2^)

**Table:**

	*x*	*y*	*z*	*U*_iso_*/*U*_eq_	
O1	−0.03069 (13)	0.6890 (3)	0.1237 (2)	0.0561 (11)	
O2	−0.07828 (14)	0.6152 (4)	−0.0343 (2)	0.0674 (14)	
O3	−0.02971 (14)	0.7095 (4)	−0.1577 (2)	0.0664 (13)	
O4	0.15588 (13)	1.0422 (4)	−0.02943 (18)	0.0528 (10)	
O5	0.15037 (13)	0.9581 (4)	0.28873 (18)	0.0574 (11)	
N1	0.15743 (14)	1.0418 (4)	0.1481 (2)	0.0418 (11)	
C1	−0.0795 (2)	0.6008 (6)	0.1437 (3)	0.0673 (19)	
C2	−0.0344 (2)	0.6874 (5)	0.0313 (3)	0.0473 (17)	
C3	0.01556 (18)	0.7776 (5)	0.0160 (3)	0.0393 (16)	
C4	0.01545 (19)	0.7844 (5)	−0.0792 (3)	0.0427 (17)	
C5	0.06228 (18)	0.8703 (5)	−0.0956 (3)	0.0461 (16)	
C6	0.10844 (18)	0.9523 (5)	−0.0193 (3)	0.0394 (14)	
C7	0.10893 (18)	0.9489 (5)	0.0764 (3)	0.0347 (14)	
C8	0.06285 (18)	0.8611 (5)	0.0931 (3)	0.0401 (16)	
C9	0.17569 (18)	1.0465 (5)	0.2465 (3)	0.0405 (16)	
C10	0.22833 (19)	1.1646 (5)	0.3026 (3)	0.0488 (16)	
C11	0.2012 (2)	1.3027 (6)	0.3419 (3)	0.071 (2)	
C12	0.2825 (2)	1.0777 (6)	0.3844 (3)	0.081 (2)	
H1	0.17846	1.10489	0.12524	0.0500*	
H1A	−0.08075	0.49015	0.12248	0.1011*	
H1B	−0.12055	0.65059	0.10854	0.1011*	
H1C	−0.06954	0.60325	0.21343	0.1011*	
H3	−0.05505	0.66231	−0.13988	0.0994*	
H4	0.15318	1.03493	−0.08643	0.0791*	
H5	0.06261	0.87259	−0.15848	0.0552*	
H8	0.06317	0.85732	0.15633	0.0484*	
H10	0.24534	1.20993	0.25653	0.0581*	
H11A	0.16985	1.36048	0.28749	0.1067*	
H11B	0.23520	1.37530	0.37963	0.1067*	
H11C	0.18139	1.25979	0.38335	0.1067*	
H12A	0.30085	0.99782	0.35606	0.1215*	
H12B	0.26605	1.02529	0.42766	0.1215*	
H12C	0.31477	1.15496	0.42134	0.1215*	

### Atomic displacement parameters (Å^2^)

**Table:**

	*U*^11^	*U*^22^	*U*^33^	*U*^12^	*U*^13^	*U*^23^
O1	0.064 (2)	0.065 (2)	0.0401 (19)	−0.0134 (17)	0.0215 (15)	0.0018 (17)
O2	0.059 (2)	0.084 (3)	0.050 (2)	−0.0224 (18)	0.0122 (16)	−0.0199 (18)
O3	0.057 (2)	0.100 (3)	0.0340 (18)	−0.0108 (19)	0.0094 (15)	−0.0240 (19)
O4	0.0585 (18)	0.080 (2)	0.0239 (15)	−0.0023 (17)	0.0207 (14)	−0.0022 (17)
O5	0.071 (2)	0.082 (2)	0.0214 (15)	−0.0249 (18)	0.0209 (14)	−0.0079 (16)
N1	0.048 (2)	0.056 (2)	0.0250 (19)	−0.0101 (18)	0.0185 (16)	−0.0026 (18)
C1	0.064 (3)	0.072 (4)	0.070 (3)	−0.017 (3)	0.031 (3)	0.005 (3)
C2	0.055 (3)	0.045 (3)	0.040 (3)	0.007 (2)	0.017 (2)	0.001 (2)
C3	0.044 (3)	0.041 (3)	0.031 (2)	0.002 (2)	0.013 (2)	−0.001 (2)
C4	0.040 (3)	0.053 (3)	0.031 (3)	0.004 (2)	0.010 (2)	−0.010 (2)
C5	0.045 (3)	0.062 (3)	0.027 (2)	0.009 (2)	0.010 (2)	−0.006 (2)
C6	0.042 (2)	0.054 (3)	0.024 (2)	0.009 (2)	0.015 (2)	0.005 (2)
C7	0.041 (2)	0.041 (3)	0.020 (2)	0.005 (2)	0.0100 (18)	0.002 (2)
C8	0.049 (3)	0.049 (3)	0.023 (2)	0.005 (2)	0.015 (2)	−0.001 (2)
C9	0.046 (3)	0.056 (3)	0.021 (2)	−0.001 (2)	0.015 (2)	−0.010 (2)
C10	0.053 (3)	0.067 (3)	0.028 (2)	−0.013 (3)	0.018 (2)	−0.009 (2)
C11	0.081 (4)	0.073 (4)	0.064 (3)	−0.018 (3)	0.034 (3)	−0.022 (3)
C12	0.057 (3)	0.103 (5)	0.068 (4)	−0.010 (3)	0.009 (3)	−0.009 (3)

### Geometric parameters (Å, º)

**Table:**

O1—C1	1.451 (6)	C7—C8	1.373 (6)
O1—C2	1.331 (5)	C9—C10	1.507 (6)
O2—C2	1.231 (5)	C10—C11	1.515 (6)
O3—C4	1.351 (5)	C10—C12	1.516 (6)
O4—C6	1.364 (5)	C1—H1A	0.9600
O5—C9	1.239 (5)	C1—H1B	0.9600
O3—H3	0.8200	C1—H1C	0.9600
O4—H4	0.8200	C5—H5	0.9300
N1—C9	1.341 (5)	C8—H8	0.9300
N1—C7	1.410 (5)	C10—H10	0.9800
N1—H1	0.8600	C11—H11A	0.9600
C2—C3	1.448 (6)	C11—H11B	0.9600
C3—C4	1.404 (6)	C11—H11C	0.9600
C3—C8	1.395 (6)	C12—H12A	0.9600
C4—C5	1.377 (6)	C12—H12B	0.9600
C5—C6	1.371 (6)	C12—H12C	0.9600
C6—C7	1.407 (6)		
			
C1—O1—C2	117.5 (3)	C11—C10—C12	112.1 (3)
C4—O3—H3	109.00	C9—C10—C11	109.8 (4)
C6—O4—H4	109.00	O1—C1—H1A	110.00
C7—N1—C9	129.7 (4)	O1—C1—H1B	110.00
C7—N1—H1	115.00	O1—C1—H1C	110.00
C9—N1—H1	115.00	H1A—C1—H1B	109.00
O1—C2—O2	120.7 (4)	H1A—C1—H1C	109.00
O1—C2—C3	114.9 (4)	H1B—C1—H1C	109.00
O2—C2—C3	124.5 (4)	C4—C5—H5	120.00
C2—C3—C4	119.2 (4)	C6—C5—H5	120.00
C4—C3—C8	119.3 (4)	C3—C8—H8	120.00
C2—C3—C8	121.6 (4)	C7—C8—H8	120.00
O3—C4—C3	122.4 (4)	C9—C10—H10	108.00
O3—C4—C5	117.4 (4)	C11—C10—H10	108.00
C3—C4—C5	120.2 (4)	C12—C10—H10	108.00
C4—C5—C6	120.1 (4)	C10—C11—H11A	109.00
C5—C6—C7	120.7 (4)	C10—C11—H11B	109.00
O4—C6—C5	123.8 (4)	C10—C11—H11C	109.00
O4—C6—C7	115.5 (4)	H11A—C11—H11B	109.00
N1—C7—C8	125.1 (4)	H11A—C11—H11C	110.00
C6—C7—C8	119.3 (4)	H11B—C11—H11C	110.00
N1—C7—C6	115.6 (4)	C10—C12—H12A	109.00
C3—C8—C7	120.6 (4)	C10—C12—H12B	109.00
O5—C9—N1	121.4 (4)	C10—C12—H12C	109.00
O5—C9—C10	122.0 (4)	H12A—C12—H12B	109.00
N1—C9—C10	116.6 (4)	H12A—C12—H12C	110.00
C9—C10—C12	110.3 (4)	H12B—C12—H12C	109.00
			
C1—O1—C2—O2	0.9 (6)	C4—C3—C8—C7	−0.1 (6)
C1—O1—C2—C3	179.9 (4)	O3—C4—C5—C6	178.3 (4)
C9—N1—C7—C6	−170.2 (4)	C3—C4—C5—C6	−1.4 (6)
C9—N1—C7—C8	11.3 (7)	C4—C5—C6—O4	−179.0 (4)
C7—N1—C9—O5	2.0 (7)	C4—C5—C6—C7	0.5 (6)
C7—N1—C9—C10	−177.9 (4)	O4—C6—C7—N1	1.4 (5)
O1—C2—C3—C4	−179.4 (4)	O4—C6—C7—C8	−179.9 (4)
O1—C2—C3—C8	−1.0 (6)	C5—C6—C7—N1	−178.1 (4)
O2—C2—C3—C4	−0.5 (7)	C5—C6—C7—C8	0.6 (6)
O2—C2—C3—C8	178.0 (4)	N1—C7—C8—C3	177.8 (4)
C2—C3—C4—O3	−0.1 (6)	C6—C7—C8—C3	−0.7 (6)
C2—C3—C4—C5	179.6 (4)	O5—C9—C10—C11	−70.1 (5)
C8—C3—C4—O3	−178.5 (4)	O5—C9—C10—C12	53.9 (6)
C8—C3—C4—C5	1.2 (6)	N1—C9—C10—C11	109.9 (4)
C2—C3—C8—C7	−178.5 (4)	N1—C9—C10—C12	−126.1 (4)

### Hydrogen-bond geometry (Å, º)

**Table:**

*D*—H···*A*	*D*—H	H···*A*	*D*···*A*	*D*—H···*A*
N1—H1···O4	0.86	2.19	2.604 (4)	109
O3—H3···O2	0.82	1.87	2.595 (4)	146
O4—H4···O5^i^	0.82	1.820	2.633 (4)	174

Symmetry code: (i) *x*, −*y*+2, *z*−1/2.
